# Multi-modal data analysis for early detection of alzheimer’s disease and related dementias

**DOI:** 10.1016/j.tjpad.2025.100399

**Published:** 2025-12-01

**Authors:** Liming Wang, Jim Glass, Lampros Kourtis, Rhoda Au

**Affiliations:** a Massachusetts Institute of Technology, Cambridge, MA, USA; b Gates Ventures, Seattle, WA, USA; c Boston University Chobanian & Avedisian School of Medicine and School of Public Health, Boston, MA, USA

**Keywords:** Dementia classification, Speech and language processing, State-space model, Multi-modal

## Abstract

Until recently, accurate early detection of clinical symptoms associated with Alzheimer’s disease (AD) and related dementias (ADRD) has been difficult. Digital technologies have created new opportunities to capture cognitive and other AD/ADRD related behaviors with greater sensitivity and specificity. Speech captured through digital recordings has shown recent promise at feasible levels of scalability because of the widespread penetration of smartphones. One such study is described in detail to illustrate the depth in which artificial intelligence (AI) analytic approaches can be used to amplify the value of audio recordings. Another modality that has also attracted research interest are ocular scans that have near term potential for validation as a digital biomarker and a point of entry for clinical care workflows. Single modality measures, however, are rapidly giving way to multi-modality sensors that are embedded in all smartphones and other internet-of-things connected devices. Artificial intelligence (AI) driven analytic approaches are able to divine clinical signals from these high dimensional digital data streams. These data driven findings are setting the stage for a future state in which AD/ADRD detection will be possible at the earliest possible stage of the neurodegenerative process and enable interventions that would significantly attenuate or alter the trajectory, preventing disease from reaching the clinical diagnosis threshold.

## 1 Introduction

With United States’ (US) Federal Drug and Administration (FDA) approval of lecanemab and donenamab to slow progression of Alzheimer's disease (AD) clinical symptoms, early diagnosis of AD has become increasingly important for initiating timely treatment, slowing disease progression, and improving patients’ quality of life and life expectancy [1,2]. But determining who to treat and when remains a significant challenge. Widespread AD in vivo pathological detection is now possible through the FDA’s more recent approvals of AD blood-based biomarkers [3]. But presence of AD pathology does not always lead to clinical expression of disease [4]. Further, clinical symptoms, particularly in the earliest stages are highly variable, resulting in a significant challenge of identifying *clinically meaningful* symptoms. Compounding the clinical detection objective is that AD is often co-morbid with other pathologies, such as vascular and/or Lewy body pathologies, which also result in cognitive and related behavioral indicators, some of which overlap with those of AD. The ethical dilemma is potential treatment of those who are AD biomarker positive but will never progress to clinically expressed disease given pharmacologically concomitant side effects. Thus, despite the exciting promise of AD blood biomarkers, the clinical utility of these blood tests will be constrained without confirmation that treatment is warranted.

Cognitive impairment is the most common clinical symptom of AD and related dementias (ADRD) and is a key primary outcome in AD clinical trials to determine intervention efficacy. Neuropsychological tests are widely used to assess cognitive state. They include multi-domain screening tests such as the widely used Mini-Mental State Examination (MMSE) [5] and domain specific assessments that typically rely on trained examiners administering standardized protocols. A comprehensive cognitive protocol is often labor-intensive to administer, comprised of tests that are culturally and educationally biased, and produce results that are variable making distinction from subtle or early-stage cognitive decline difficult to discern [6]. Manual scoring approaches also may miss nuanced indicators that signal cognitive impairment. Technological advances have emerged that can alleviate these issues. Digital capture of behaviors through smartphones, wearables and other internet of things (IoT) present an opportunity to overcome the limitations of standardized neuropsychological testing and do so at a scale that has not been hereto for possible.

In this perspective, we highlight two areas in which digital technologies are being used in AD/ADRD because of their non-invasive and feasibly scalable nature. The first section provides a detailed description of research being done with speech as an illustration of how both technological advances are being applied to both data collection and analysis. The second section summarizes ocular scans research to introduce the potential realm of digital biomarkers. The final section looks beyond these highlighted efforts to present a vision of the future, well beyond the single modality approach and where much scientific discovery remains to be done.

## 2 Speech as a measure of early AD related symptoms

Many IoT devices have speech recording capacity and because speaking is a cognitively complex task, in the context of cognitive impairment detection have already led to automatic dementia classification (ADC) systems that infer cognitive state directly from digitally recorded speech of neuropsychological tests [[6], [7], [8]]. The potential of analyzing speech as an alternative approach to assessing cognitive state is particularly intriguing because it taps into multiple cognitive domains to produce intelligible content and most people speak, regardless of their education, culture, sex, or language.

Individuals with AD and other forms of dementia exhibit measurable changes in their speech production, seen in both the acoustic domain and the language domain [9]. These changes often precede noticeable cognitive related symptoms [10]. Digital recordings of structured speech, such as from neuropsychological testing, also allow for concurrent validation of speech markers as a surrogate measure of cognition compared to neuropsychological tests [11].

ADC systems that analyze speech recordings aim to detect subtle linguistic and paralinguistic cues (e.g., hesitations, disfluencies, semantic anomalies) indicative of a neurodegenerative disorder. In addition, these systems can also mitigate well-known test question biases [8,12,13] because the speech analysis can analyze all content and is not limited to scoring parameters that are impacted by test item relevance. Early work on ADC tasks for AD detection (ADD) used classical machine learning algorithms with hand-crafted speech and linguistic features [7,14]. More recent systems leverage deep learning architectures such as convolutional [11] and recurrent [15] neural networks and neural embeddings from pretrained speech representation models such as wav2vec 2.0 [16,17] and Whisper [18,19] as well as text language models [18,20]. These speech processing methods have led to published studies that evidence the power of speech analysis across the dementia progression spectrum. Fraser et al. (2016), using a computational approach with 370 linguistic and acoustic features, achieved up to 82 % accuracy in classifying AD versus controls from picture descriptions [21]. Eyigoz et al demonstrated that linguistic variables derived from a pre-recorded picture description task could predict future AD onset (almost 15 years in advance) from a cognitively normal baseline with a significant area under the curve (AUC) of 0.74 and an accuracy of 0.70 [22]. Pan et al , exploring different automatic speech recognition (ASR) paradigms and bidirectional encoder representations from transformers (BERT)-based classification from the DementiaBank 2021 publicly available audio only speech dataset called Alzheimer's Dementia Recognition through Spontaneous Speech (ADReSS). They reported test results for their best acoustic-only model at 74.65 % accuracy and their best linguistic-only model at 84.51 % accuracy [23]. García-Gutiérrez et al. (2024), leveraging paralinguistic (acoustic) features combined with sociodemographic data, showed the ability to identify individuals with AD (F1-score = 0.92) and MCI (F1-score = 0.84). They found differentiating MCI from SCD (Subjective Cognitive Decline) yielded an AUC of 0.80, and MCI from AD had an AUC of 0.73 [24]. Agbavor and Liang (2022) found that GPT-3 based text embeddings notably outperformed conventional acoustic feature-based approaches for AD classification [25]. Their model achieved 80.3 % accuracy, 72.3 % precision, 97.1 % recall, and an 82.9 % F1-score on the ADReSSo unseen test set. For Mini Mental State Exam score prediction, a short dementia screening assessment, they found that a Ridge regression model (RMSE) using acoustic features had an RMSE of 6.250, while their GPT-3 Babbage model achieved a lower RMSE of 5.163. Heitz et al. (2024) leveraged GPT-4 to extract five semantic features from spontaneous speech transcripts with up to 93.1 % accuracy on manual transcripts and 90.5 % on ASR transcripts [26]. Amini et al. (2024) used natural language processing (NLP) and machine learning (ML) techniques on recorded neuropsychological test interviews to predict progression from MCI to AD within six years and achieved an accuracy of 78.5 % and a sensitivity of 81.1 %, with a moderate specificity of 75 % [27].

Despite progress in algorithmic design, existing work still focuses on sentence-level speech segments and small datasets such as ADReSS [6,14] and the Framingham Heart Study [7]. Their study surprisingly found that AD is possible with examiner speech only, indicating examiner bias in administration of standardized neuropsychological tests [8,12,13] that are presumed to follow prescriptive administration protocols . Existing speech-specific ADC systems are fundamentally limited in their ability to process neuropsychological test recordings that are typically long in duration, with many published protocols taking 1+ hours to administer. This constraint often forces segment-level inference using forced alignment or manual heuristics [7,17,18], leading to context fragmentation and a drop in fine-grained classification performance [28].

To date, many voice-related analyses have focused on acoustic features. The advantage is acoustic features can be easily extracted regardless of native language spoken. Available open-source automatic speech recognizer (ASR) such as Whisper have led to transcriptive based pipelines that can utilize natural language processing for analysis, but ASR accuracy is more variable outside of the most commonly spoken languages (e.g., English, Spanish, etc.). ASR suffer from loss of acoustic information as well as error propagation, especially in noisy, spontaneous and multi-speaker conversational settings, whereas acoustics only suffer from loss of language information, such as word choices, sentence structure and content richness. Further, speech generated in a natural context is generally longer in length and involves exchange between two or more speakers. To address the challenges of analyzing long-length digital recordings that include interactive speech, we have proposed leveraging state-space models (SSMs) [29,30], a family of architectures designed for efficient long-sequence modeling.

## 3 Joint acoustic and linguistic analysis of interactive long-length speech recordings

SSMs scale linearly in both space and time, making them ideal for modeling longer-length speech recordings without segmentation. For example, the dementia information in recording of neuropsychological test administration can be subtle and sporadic, with many conversational turns offering little diagnostic value [12]. SSMs’ natural capacity for temporal compression allows them to distill salient patterns with minimal information loss, making them particularly well-suited for the ADC task. Below, we present *Demenba* [31], a memory- and compute-efficient architecture trained on over 1000 h of neuropsychological tests with balanced representation across dementia stages.

**Fig. 1:**
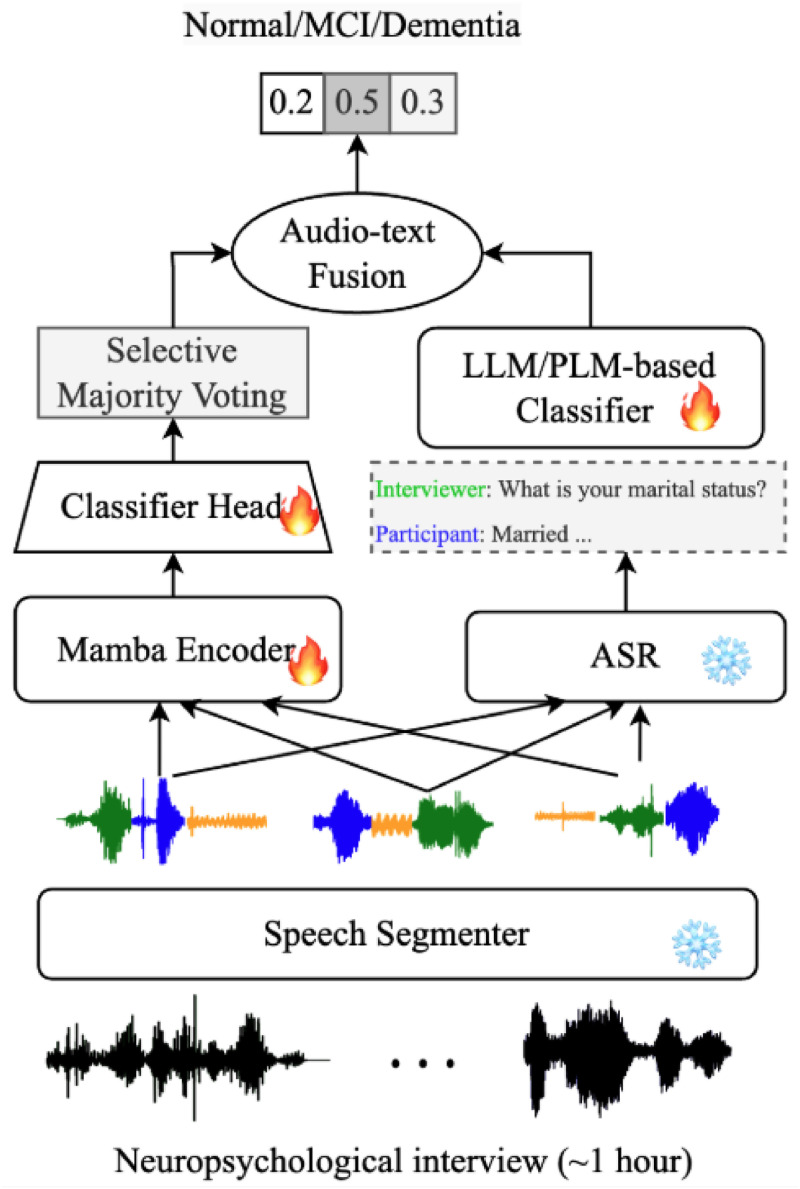
Overall architecture of the proposed ADC model. The frozen speech segmenter divides the hour-long recording into shorter segments, a trainable SSM-based audio classifier and a trainable text- based text classifier. The predictions from the two classifiers are then combined via late fusion.

Fig. 1 shows the overall design of our multimodal dementia classification system, which combines both speech and language analysis. The system consists of four main components: (1) a speech segmenter, (2) an automatic speech recognizer (ASR), (3) an audio-based dementia classifier, and (4) a text-based dementia classifier.

## 4 Audio analysis

The **speech segmenter** takes an hour-long neuropsychological test recording and breaks it into shorter, more manageable segments. It separates examiner speech, participant speech and pauses. This not only makes the system more efficient but also allows us to study how factors such as silence duration or examiner/participant speaking turns affect dementia classification.

Next, the **audio dementia classifier** evaluates how the participant sounds. It uses an advanced deep learning model [30,[32], [33], [34], [35]] to capture long-range dependency in the speech. Each segment is assigned a probability of belonging to a dementia category. Since dementia-related cues may appear only in certain moments, the system highlights the most informative segments and weighs them more heavily in the final decision.

## 5 Text analysis

To complement the audio, the text dementia classifier looks at what the participant says. The ASR system, Whisper [19], automatically transcribes the entire recording into text. A large language model (LLM) [36,37] or pretrained language model (PLM) [38] then analyzes the transcripts, assessing whether the participant’s responses are coherent, accurate, and consistent with normal cognition. Beyond simply assigning a diagnosis, the LLM can also generate a clinician-friendly narrative summary, improving interpretability for medical use.

## 6 Decision fusion

Lastly, the system integrates the predictions from both audio and text branches to determine overall dementia severity. This combined approach improves accuracy compared to using speech or text alone. In particular, for 2-class classification, the AUC of the best text-only and audio-only approaches are 91 % and 87 %, while the combined approach achieves an AUC of 95 %. The reason for this synergy may be that audio models tend to capture prosodic cues such as hesitation and intonation, whereas text models tend to capture lexical/linguistic patterns like filler words and semantic incoherence.

## 7 Clinical evaluation

We tested the system on the Framingham Heart Study (FHS) dataset [12], which includes about 11,000 h-long neuropsychological test recordings, 2058 of which have been adjudicated and labeled as cognitively intact (*n* = 936) and cognitively impaired (*n* = 1122). For 3-class fine-grained classification task, we further divide the cognitively impaired class into mild cognitive impairment (MCI) and dementia classes.

In the two-class setting (normal vs. dementia), our system (Demenba-medium) achieved an area under curve (AUC) of 92 %, surpassing a 6 % improvement over prior methods. For the three-class task (intact, MCI, dementia), performance remained strong with an AUC of 83 %, a 14 % AUC gain over the previous state of the art. Importantly, the advantage of our method grew when distinguishing more subtle differences between MCI and dementia, suggesting that the system is particularly sensitive to early signs of cognitive decline. Our method consistently outperformed prior methods, with the largest gains observed in the more challenging 3-class setting. Importantly, the system scales effectively, handling over 1000 h of training data and reliably analyzing speech segments up to 6 min long — capabilities essential for real-world clinical recordings. Details about the methods described above are provided in Wang et al. [31].

## 8 Concluding remarks regarding speech

Our Demenba analyses highlight the complementary roles of acoustic patterns (how someone speaks), speaker dynamics (who is speaking), and linguistic content (what is said) in dementia classification. These findings provide new insights into how different aspects of speech and language reflect cognitive status.Looking ahead to enhancing the potential of speech, we aim to enhance both the performance and interpretability of our models by integrating them with multimodal LLMs, which can combine speech, language and other clinical as well as digital data. Another challenge is to improve the accuracy of our approach by handling data variability introduced by factors such as accent, gender, age and additional health conditions.

In addition to the publicly available DementiaBank ADReSS, the emergence of other speech datasets such as that from the Alzheimer’s Drug Discovery Foundation SpeechDx, a multi-center, longitudinal study that is collecting longitudinal voice recordings from approximately 2000 participants with strong clinical characterization profiles provide resources from which to accelerate the translation of audio recording research to clinical utility. Future directions enabled by these datasets include studying finer-grained classification of dementia subtypes and generalization performance on other dementia datasets, to ensure broader clinical applicability.

At the same time, the increasing diversity of datasets underscores important challenges the field must address. Harmonization across sites and recording protocols is critical to ensure that models trained on one cohort remain valid across others. Equally important are anonymization and de-identification strategies, which safeguard participant privacy while retaining the clinical richness of speech data. Developing methods that balance privacy with utility, while also addressing variability in recording conditions, accents, and disease progression, will be essential for making speech-based biomarkers both reliable and ethically deployable in real-world healthcare.

Further, while our method is a step toward more fine-grained categorization of dementia, it remains to be tested whether our method can be extended to detect more subtle cues of dementia in the early stages of dementia, which are much more challenging than differentiating MCI and AD. More clinically relevant metrics such as the RMSE of predicting the MMSE score can also be assessed, provided that the ground truth scores are available.

The broad penetration of smartphones provides a ubiquitous tool for capturing and processing natural speech during everyday phone conversations, offering a rich and passive means of collecting longitudinal speech and language data. This continual, real-world sampling enables for the detection of subtle changes in vocal, lexical, and syntactic patterns and identify early signs of cognitive decline if used over time.

## 9 Beyond speech: eye as a window to the brain and as a potential digital biomarker

Digital ocular image instruments can track eye movements, the analysis of which is playing an increasingly significant role in AD research due to its potential as a non-invasive tool for early detection and monitoring of the disease. However, like all other cognitive domain testing, the study of eye movements relies on the respective stimuli context such as smooth pursuit, scene viewing, visual search and other. Amongst the various stimuli, reading is a well-defined task that occurs numerous times per day on mobile devices without any prompt. The average person who is literate reads 3000–10,000 words per day on their mobile device. Reading metrics are a highly applicable biomarker in AD research due to the reading process’ standardized nature as a well-defined, complex cognitive process whose underlying mechanisms are profoundly impacted by early AD-related cognitive and neurological changes, resulting in quantifiable alterations in eye movement patterns such as increased gaze duration, more fixations, and a loss of the contextual predictability effect.

For those who are literate and are not visually impaired, reading is a complex cognitive activity that requires the fine integration of attention, ocular movements, word recognition, language comprehension, working memory, and semantic memory. Many of these cognitive processes, such as attention, inhibitory control, working memory, and decision-making, have been well-documented to be impaired in the early stages of AD/ADRD. Subtle alterations in movement coordination and planning, which are often unnoticed in early AD/ADRD when performing other fine motor tasks like writing, can be precisely detected through eye movement analysis during reading. This is because neurological connectivity changes occur early in the course of AD, disrupting controlled information processing that is critical for reading.

## 10 Previous studies demonstrating validity of ocular movements as a potential AD biomarker

The eye offers an intriguing opportunity to explore the concept of digital indices as digital biomarkers. The advantage of ocular research is the finite and well-defined measurements and the transparency in analysis. To move from a digital measure to a digital biomarker requires validation that can be easily reproduced or replicated. The few studies summarized below evidence the type of results that have much more direct clinical translation, which is necessary for FDA approval as a biomarker. There is also an existing clinical pathway for conducting eye scans, which would allow more easy integration of an ocular biomarker into the clinical workflow.

**Fig. 2:**
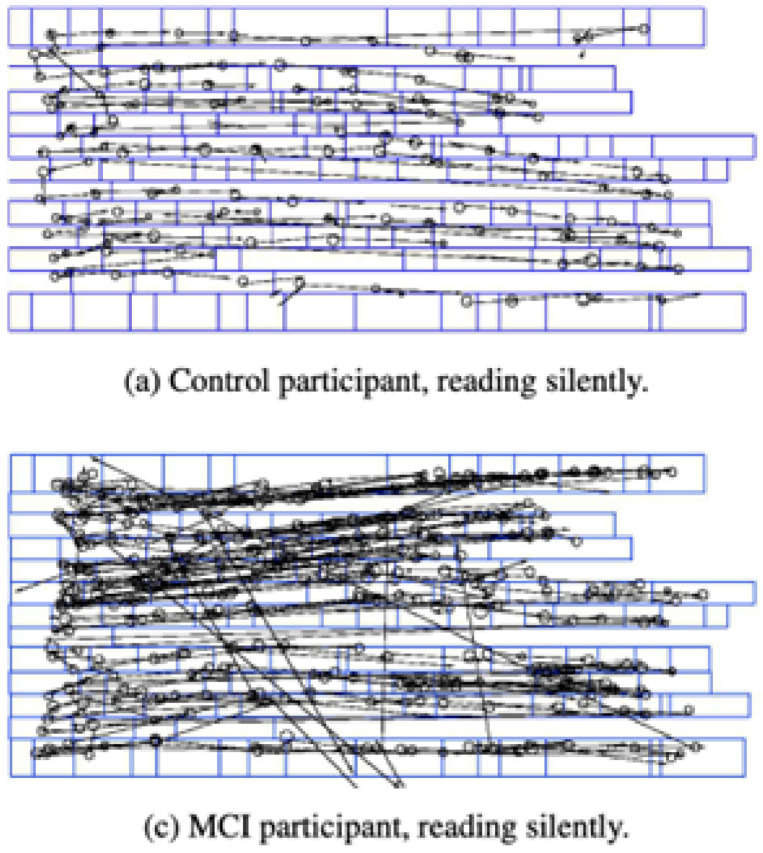
Reproduced from Fraser et al. 2017, showing less scattered single fixations and outgoing saccades during silent reading from (a) control compared to (c) MCI participants.

A series of studies have been able to show that ocular measures are able to capture in a quantifiable manner, cognitively related natural behaviors, such as reading. In an early study, Fernandez et al. 2013 found a sizeable difference of 23 % in outgoing saccade size between AD and Controls [39]. In another study, Fernandez et al. found that participants with mild AD did not show reduced gaze duration when reading highly predictable text as compared to cognitively intact controls, suggesting early impairments in memory-related mechanisms that support contextual word processing [40]. In another follow up study, Fernandez et al. 2015) further validated that participants with mild AD exhibited significantly more total, first-pass, and especially second pass fixations compared to cognitively healthy controls, indicating increased rereading behavior during both regular and high-predictability sentence reading [41]. They also showed fewer single fixations and shorter outgoing saccades, suggesting impaired contextual word processing (see Fig. 2). In general, cognitively healthy readers adjust their gaze based on the predictability of preceding and upcoming words, while those with AD do not, reflecting early deficits in semantic anticipation and memory-guided eye movement control.

Biondi et al. 2018 reported performance of a softmax classifier using a series of features like first pass fixations, unique fixations and multiple fixations Refixations was 88.7 % to classify Controls and 91.0 % for AD patients [42]. Taken together, these studies show that ocular measures can differentiate with specificity between those with and without AD. These results are illustrative of the types of ocular studies underway that lend credence to the eventual validation of digital ocular AD biomarkers.

## 11 Digital interactions

Given that over 6.8 billion people are estimated to use a smartphones [43] it is worth emphasizing the unique opportunity they provide for continuous remote monitoring of AD/ADRD related behavioral changes. Embedded within each smartphone are the multiple sensors described above that can collect the raw 3-dimensional digital data streams that AI driven algorithms are interpreting into behavioral measures. Patterns of sensor rhythms reflect executive function, sleep-wake stability, and behavioral regularity, all of which deteriorate with mild cognitive impairment [44]. Kaye et al., (2011) used activity sensors and digital markers that included phone use to track daily regularity as a proxy for cognitive decline [45]. Previous studies have demonstrated that frequent app switching and short dwell times can reflect impulsivity or distractibility while reduced diversity may reflect narrowing interests or cognitive fatigue [46]. Changes in session structure can reflect attention span, fatigue, or executive function breakdown. Slower typing and higher error rates may correlate with motor or processing issues [47]. These studies in the aggregate provide further evidence that cognitive decline is associated with disrupted routines or decreased behavioral regularity, which can all be measured through sensors embedded in every smartphone [48].

## 12 Multi-Modal: the next frontier

Despite the promise of speech and ocular biomarkers, limiting early detection of AD/ADRD clinical symptoms to a single modality would be short-sighted. Cognition is reflected in virtually all bodily movement. Sensors embedded in smartphones, wearables and in home devices also collect behavioral movements. The different sensor modalities in combination, provide a comprehensive multi-modal assessment platform from which to detect early changes in cognitive and other related behaviors. Further, multi-modal assessments can help circumvent limitations of any single modality measurement. For example, despite the promise of reading-based ocular biomarkers, there are multiple factors beyond literacy levels that can impact accuracy of measurement including educational attainment levels, baseline or concomitant decline in visual acuity from cataracts, glaucoma, macular degeneration or other age-related eye disorders, whether reading materials are in the person’s native language, language, etc. Other comorbid health conditions such as hearing loss, musculoskeletal related problems, breathing difficulties, are other examples of factors that can impact data collected from any single digital format, Successful interpretation of high volume and highly variable fluctuating patterns in different person-specific combinations of digital data will likely lead to much more accurate differentiation between normal behavioral fluctuations from those that are reflective of early neuropathological progression. While previous work centered on single sensor modality accounts for much of the current digital adoption into clinical research, trials and care, an inflection point is nearing where there will be a shift to multi-dimensional data streams uniquely customized to the individual, but fueled largely by AI analytic methods that are still able to effectively extract common meaningful information from them. If these AI solutions result in high sensitivity and specificity for AD/ADRD at much lower cost and far greater reach, they will accelerate the digital revolution that is already underway.

Other issues regarding digital biomarkers that needs significant consideration are the validation process and the transition to clinical care. Acquisition of data through digital devices does not automatically mean the resulting measurement is a “digital biomarker”. Neither digital voice or eye movements can yet be considered digital biomarkers within the US until they go through the same rigorous validation process that both AD imaging and plasma biomarkers have gone through following FDA guidelines, which includes specific context of use [49]. Further FDA approval alone will not lead to clinical use in the US. Many factors impact the path post-validation such as whether the test is widely accessible, whether test results are easily interpretable or whether clear treatment guidelines are available, particularly when specialized expertise is unavailable [50]. Advances in research that capitalize on approaches that are ubiquitously obtainable can ensure this trend does not have to continue, despite the rise in the number of dementia cases around the world,. Combination therapies that are increasingly being touted [51], comprised of both pharmacologic and non-pharmacologic interventions, could have potential to disrupt the trajectory of AD/ADRD progression to the point that disease symptoms at the clinical diagnosis threshold is never reached. But this vision is contingent on detecting those changes many years if not decades earlier in the insidious onset process. Through smartphones and other IoT devices, single modality digital assessment tools are rapidly giving way to multi-modal ones. AI innovations today may soon be replaced by an even more powerful quantum computing environment. While much is unknown, what is certain is that technological advances are solving the challenge of early detection of AD/ADRD to the entire at risk global population, bringing with it great optimism in eradicating them.

## Consent statement

Consent was not necessary for the purposes of this perspective piece.

## CRediT authorship contribution statement

**Liming Wang:** Writing – review & editing, Writing – original draft. **Jim Glass:** Writing – review & editing, Writing – original draft. **Lampros Kourtis:** Writing – review & editing, Writing – original draft, Conceptualization. **Rhoda Au:** Writing – review & editing, Writing – original draft, Conceptualization.

## Declaration of competing interest

The authors declare the following financial interests/personal relationships which may be considered as potential competing interests: Rhoda Au reports a relationship with Novo Nordisk Inc that includes: consulting or advisory, speaking and lecture fees, and travel reimbursement. Rhoda Au reports a relationship with Signant Health that includes: consulting or advisory. If there are other authors, they declare that they have no known competing financial interests or personal relationships that could have appeared to influence the work reported in this paper.

## Glossary of Key Terms

Automatic Dementia Classification (ADC): Computational systems that detect dementia from digital signals such as speech recordings.
Automatic Speech Recognition (ASR): Technology that converts spoken language into text.
Paralinguistic Features: Non-verbal aspects of speech such as pitch, pauses, hesitations, or intonation.
Linguistic Features: Verbal aspects of speech, including vocabulary, syntax, coherence, and semantics.
Deep Learning: Neural network-based machine learning models used for automated feature extraction and classification.
Convolutional Neural Network (CNN): A deep learning architecture effective for analyzing acoustic speech signals.
Recurrent Neural Network (RNN): A sequence-processing deep learning model used for temporal data like speech
State-Space Models (SSMs): Architectures designed for efficient modeling of long sequences, applied here for long speech recordings.
Language Models (LMs): Neural models trained on text data to capture linguistic patterns and meaning.
Area Under the Curve (AUC): A measure of a model’s ability to distinguish between classes.
